# Impact of HIV on the Oral Microbiome of Children Living in Sub-Saharan Africa, Determined by Using an *rpoC* Gene Fragment Metataxonomic Approach

**DOI:** 10.1128/spectrum.00871-23

**Published:** 2023-07-10

**Authors:** Allison E. Mann, Lauren M. O'Connell, Esosa Osagie, Paul Akhigbe, Ozoemene Obuekwe, Augustine Omoigberale, Colton Kelly, the DOMHaIN Study Team, Modupe O. Coker, Vincent P. Richards

**Affiliations:** a Department of Biological Sciences, Clemson University, Clemson, South Carolina, USA; b Institute of Human Virology Nigeria, Abuja, Nigeria; c University of Benin Teaching Hospital, Benin, Edo State, Nigeria; d Department of Oral Biology, Rutgers School of Dental Medicine, Rutgers University, Newark, New Jersey, USA; e School of Dentistry, University of the Pacific, San Francisco, California, USA; Nanchang University

**Keywords:** HIV, *Streptococcus mutans*, dental caries, HIV exposure, bacteria, oral microbiome

## Abstract

Children living with HIV have a higher prevalence of oral diseases, including caries, but the mechanisms underlying this higher prevalence are not well understood. Here, we test the hypothesis that HIV infection is associated with a more cariogenic oral microbiome, characterized by an increase in bacteria involved in the pathogenesis of caries. We present data generated from supragingival plaques collected from 484 children representing three exposure groups: (i) children living with HIV (HI), (ii) children who were perinatally exposed but uninfected (HEU), and (iii) unexposed and therefore uninfected children (HUU). We found that the microbiome of HI children is distinct from those of HEU and HUU children and that this distinction is more pronounced in diseased teeth than healthy teeth, suggesting that the impact of HIV is more severe as caries progresses. Moreover, we report both an increase in bacterial diversity and a decrease in community similarity in our older HI cohort compared to our younger HI cohort, which may in part be a prolonged effect of HIV and/or its treatment. Finally, while Streptococcus mutans is often a dominant species in late-stage caries, it tended to be found at lower frequency in our HI cohort than in other groups. Our results highlight the taxonomic diversity of the supragingival plaque microbiome and suggest that broad and increasingly individualistic ecological shifts are responsible for the pathogenesis of caries in children living with HIV, coupled with a diverse and possibly severe impact on known cariogenic taxa that potentially exacerbates caries.

**IMPORTANCE** Since its recognition as a global epidemic in the early 1980s, approximately 84.2 million people have been diagnosed with HIV and 40.1 million people have died from AIDS-related illnesses. The development and increased global availability of antiretroviral treatment (ART) regimens have dramatically reduced the mortality rate of HIV and AIDS, yet approximately 1.5 million new infections were reported in 2021, 51% of which are in sub-Saharan Africa. People living with HIV have a higher prevalence of caries and other chronic oral diseases, the mechanisms of which are not well understood. Here, we used a novel genetic approach to characterize the supragingival plaque microbiome of children living with HIV and compared it to the microbiomes of uninfected and perinatally exposed children to better understand the role of oral bacteria in the etiology of tooth decay in the context of HIV exposure and infection.

## INTRODUCTION

Dental caries is the most common chronic oral disease globally, affecting an estimated 2 billion people worldwide, 520 million of which are children (1). Untreated dental caries can be painful, delay speech development, and lead to nutritional deficiencies, poor educational performance, and an overall lower quality of life (2, 3). The etiology of caries involves the proliferation of acidogenic and acidophilic bacteria in the oral cavity (typically Streptococcus mutans and other low-pH Streptococcus species), the effects of which are exacerbated by lack of access to oral health care and cariogenic diets high in sugars and fermentable carbohydrates (4–8).

People living with HIV frequently experience chronic oral health problems, including oral candidiasis, periodontitis, gingivitis, chronic dry mouth, and oral hairy leukoplakia (9–11). Recent research has demonstrated that along with other oral diseases, children living with HIV have a higher prevalence of caries in primary and permanent dentition, which is associated with viral loads and immune status (12–14). However, the mechanisms explaining this higher caries burden or risk in children living with HIV, particularly in sub-Saharan Africa, are not fully understood. Given the importance of host immunity in modulating the oral microbiome (15, 16) and the reported higher frequency of caries in children living with HIV, we expect that the community of oral bacteria detected in supragingival plaque will be more cariogenic than those found in children who were unexposed to the virus.

In the current study, we used a novel gene fragment metataxonomic approach targeting an approximately 478-bp region of the bacterial gene *rpoC* to survey the supragingival plaque microbiomes of 484 Nigerian children representing three exposure groups: (i) children living with HIV, i.e., HIV infected (HI); (ii) children who had been perinatally exposed to HIV but uninfected, i.e., HIV exposed but uninfected (HEU); and (iii) children who had no exposure to HIV, i.e., HIV unexposed and uninfected (HUU). We included children that were perinatally exposed but uninfected in our study design, as a substantial body of literature has demonstrated that exposure to the virus or antiretroviral therapy (ART) *in utero* has systemic health effects, including impaired growth outcomes, an increased susceptibility to infections, and increased infant mortality compared to uninfected and unexposed children (17–23). We aimed to characterize and compare the supragingival microbiotas of these groups of children across six progressive stages of caries and hypothesized that samples from HI children would reflect a more acidogenic/cariogenic community than the other groups. Our novel metataxonomic approach documented an underappreciated diversity among bacterial residents of supragingival plaque and highlights our limited current understanding of the ecological role of many members of the oral community.

## RESULTS

We collected a total of 882 supragingival plaque samples from 564 children between May and December of 2019 at the University of Benin Teaching Hospital (UBTH), Benin City, Nigeria, as part of the Dental Caries and its Association with Oral Microbiome and HIV in Young Children—Nigeria (DOMHaIN) Study (24). Each plaque sample was collected from a single tooth to identify intraindividual variation in the supragingival plaque microbial community. First, plaque samples were categorized according to the condition of the tooth of origin using the International Caries Detection and Assessment System (ICDAS) (25), as follows: (i) a plaque collected from a healthy tooth with no cavity or lesion present (H; ICDAS score = 0), (ii) a plaque collected from a tooth with an active enamel carious lesion (E; ICDAS score = 1 to 3), or (iii) a plaque collected from a tooth with an active dentin carious lesion (D; ICDAS score ≥ 4) (26). Next, the overall oral health of the child was characterized by their observed caries experience: (i) caries free (CF), i.e., no clinical or reported evidence of caries (number of decayed, missing, and filled teeth [DMFT] = 0); (i) caries active with only enamel lesions present (CE; DT = 0, MFT ≥ 0); or (iii) caries active with carious lesions in the dentin of at least two teeth (CD; DT ≥2, MFT ≥ 0). Individual samples were therefore placed in one of six progressive disease states, as follows: (i) a healthy tooth collected from a child with no caries (H-CF), (ii) a healthy tooth collected from a child with active enamel caries (H-CE), (iii) a healthy tooth collected from a child with active dentin caries (H-CD), (iv) a tooth with an active enamel cavity from a child with active enamel cavities (E-CE), (v) a tooth with an active enamel cavity from a child with active dentin cavities (E-CD), and (vi) a tooth with an active dentin cavity from a child with active dentin cavities (D-CD). Our nested classification scheme for tooth and oral health is presented in Table 1. Detailed metadata for each sample included in this study can be found in Table S1 in the supplemental material. We found that HI children in this cohort had a higher frequency of severe caries (D, 18%; E, 13%) than HEU (D, 12%; E, 4%) and HUU (D, 10%; E, 10%) children. Figure S1 shows the distribution of samples (*n* = 748) by study group, overall oral health status, and tooth health status.

**TABLE 1:** Nested tooth and oral health classification scheme^*a*^

Individual tooth health	Participant oral health	Individual tooth health + participant overall oral health
Healthy (H)	Caries free (CF)	Healthy tooth from caries-free mouth (H-CF)
	Enamel caries present (CE)	Healthy tooth from mouth with active enamel caries (H-CE)
	Dentin caries present (CD)	Healthy tooth from mouth with active dentin caries (H-CD)
Enamel cavity present (E)	Enamel caries present (CE)	Tooth with enamel lesion from mouth with active enamel caries (E-CE)
	Dentin caries present (CD)	Tooth with enamel lesion from mouth with active dentin caries (E-CD)
Dentin cavity present (D)	Dentin caries present (CD)	Tooth with dentin cavity from mouth with active dentin cavities (D-CD)

a Each sample was classified according to the individual tooth health and overall participant oral health at the time of sampling into one of six progressive disease states.

**FIG 1:**
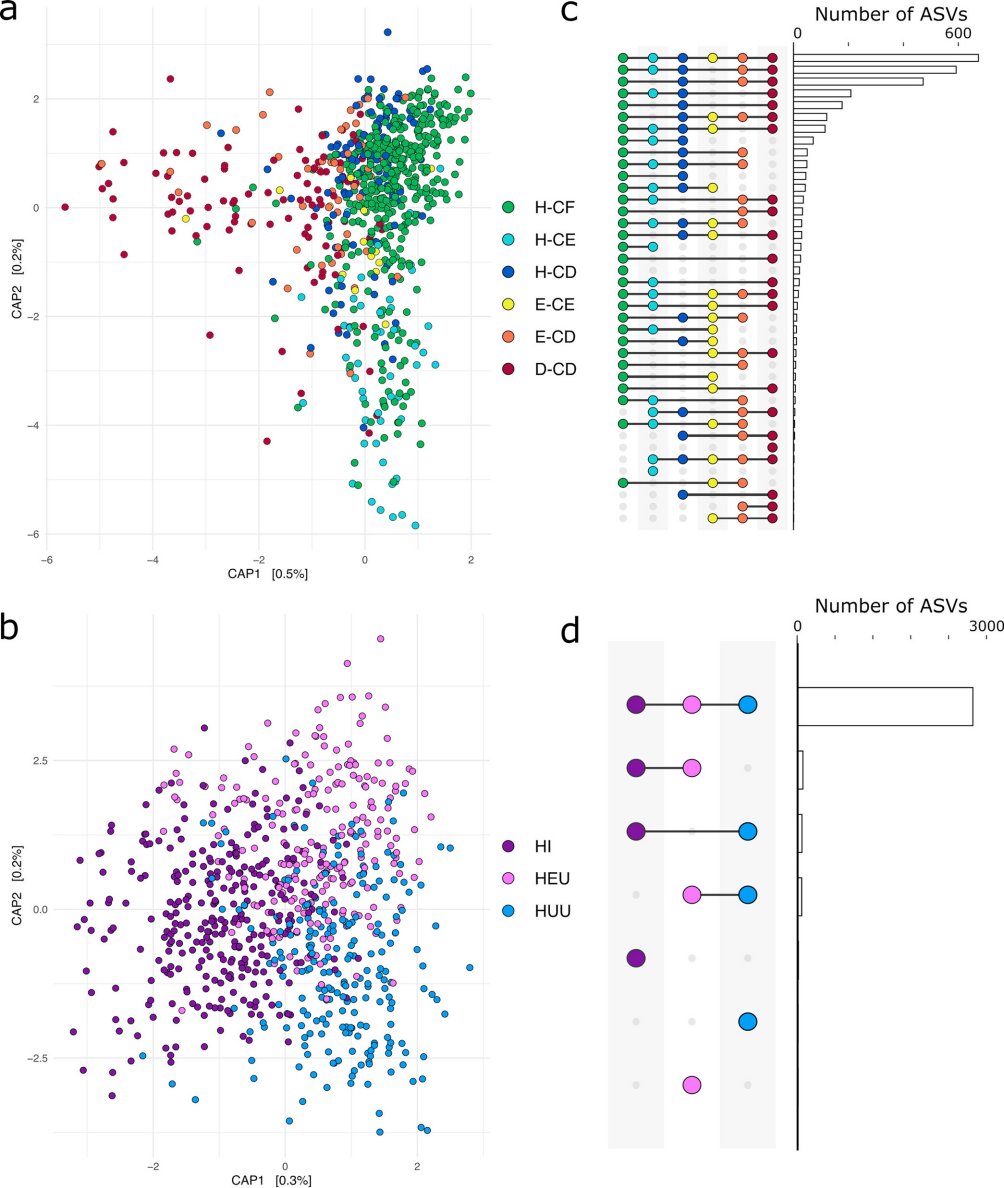
HIV status and tooth health are the main drivers of microbial community structure and diversity. (a) Capscale plot of bacterial beta diversity as measured by a Bray-Curtis dissimilarity matrix colored by specific tooth health category. (b) Capscale plot of bacterial beta diversity as measured by a Bray-Curtis dissimilarity matrix colored by HIV status. (c) Shared and unique ASVs between plaque samples collected from teeth of different health states. (d) Shared and unique ASVs between HIV categories.

On average, we generated 34,736 raw *rpoC* amplicon reads per sample (standard deviation [SD], 24,346) and an average of 25,910 (SD, 21,135) were retained after quality filtering (Table S2). After quality filtering, taxonomic assignment, and removing any amplicon sequencing variants (ASVs) with a prevalence threshold of less than 1% across the total data set, we were left with 2,969 unique ASVs. Tooth health was the most significant driver of bacterial variation, as measured by permutational multivariate analysis of variance (PERMANOVA) (*R*^2^ = 0.02, *P* = 0.001), followed by age (*R*^2^ = 0.01, *P* = 0.001), HIV status (*R*^2^ = 0.006, *P* = 0.001), and sex (*R*^2^ = 0.002, *P* = 0.03). We found no significant differences in alpha diversity (observed ASVs and Shannon diversity) by either HIV status or tooth health group (Fig. S2). Next, we performed beta diversity ordination analyses grouped by either HIV status or oral health. As the total variance explained by unsupervised ordination methods is low, as measured by weighted UniFrac analysis (Fig. S3a and b), we generated capscale plots using a distance-based redundancy analysis approach (27), which allows us to ground our dissimilarity analysis by variables of interest. Ordination of beta diversity plots was constrained by sample groupings, either by HIV group (Fig. 1a) or tooth health (Fig. 1b). In this way, response variables that contribute a small amount of the total community variance (indicated as percentages on the axes) can be visualized. Results of this analysis indicate that teeth without active carious lesions tend to have more similar microbial communities independent of the overall oral health of the individual, while those with active carious lesions tend to be distinct (Fig. 1a). To better understand the predictive power of sample metadata categories for the composition of the oral microbiome, we next performed random forest classification analysis using 10,000 trees, with the expectation that the level of classification accuracy reflects the level of taxonomic distinctiveness of communities among categories. For example, high prediction accuracy would reflect strong community distinctiveness, whereas low accuracy would reflect weak distinctiveness (i.e., less differentiation among communities). This approach complements the Bray-Curtis dissimilarity metric by providing an additional perspective on community differentiation. As random forest analyses are heavily influenced by imbalanced sample size, we only considered analyses performed between 100 randomly sampled H-CF, H-CD, and D-CD samples. Healthy teeth in caries-free mouths (H-CF) and teeth with late-stage dentin caries (D-CD) were the most distinctive in this analysis and were rarely misclassified as one another. For example, H-CF samples were correctly identified in 67% of all cases and misclassified as D-CD in only 11% of cases, while D-CD samples were correctly identified in 65% of cases and H-CF samples in only 9% of cases. Interestingly, H-CD samples are intermediate between the two health extremes, with 38% correctly identified as H-CD, 39% misclassified as H-CF, and 23% misclassified as D-CD. Full results of this analysis and a confusion matrix can be found in Table S3.

Like tooth health, HIV status was also a significant (albeit weak) driver of the microbial community, and plaque samples collected from HI, HEU, and HUU children formed three distinct yet overlapping groups. Plaque samples collected from HEU and HUU children tended to be more similar than those from HI children, but only a small proportion of the total observed variance was explained by these groupings alone (Fig. 1b). However, plaque samples collected from children living with HIV were correctly identified using random forest classification in 88% of cases, while correct prediction occurred in only 45% of plaque samples originating from HEU children and in only 31% of plaque samples originating from HUU children. Interestingly, plaque samples collected from both HEU and HUU children were most often misclassified as those originating from HI children (39% and 31%, respectively) (full random forest results and a confusion matrix can be found in Table S4).

**FIG 2:**
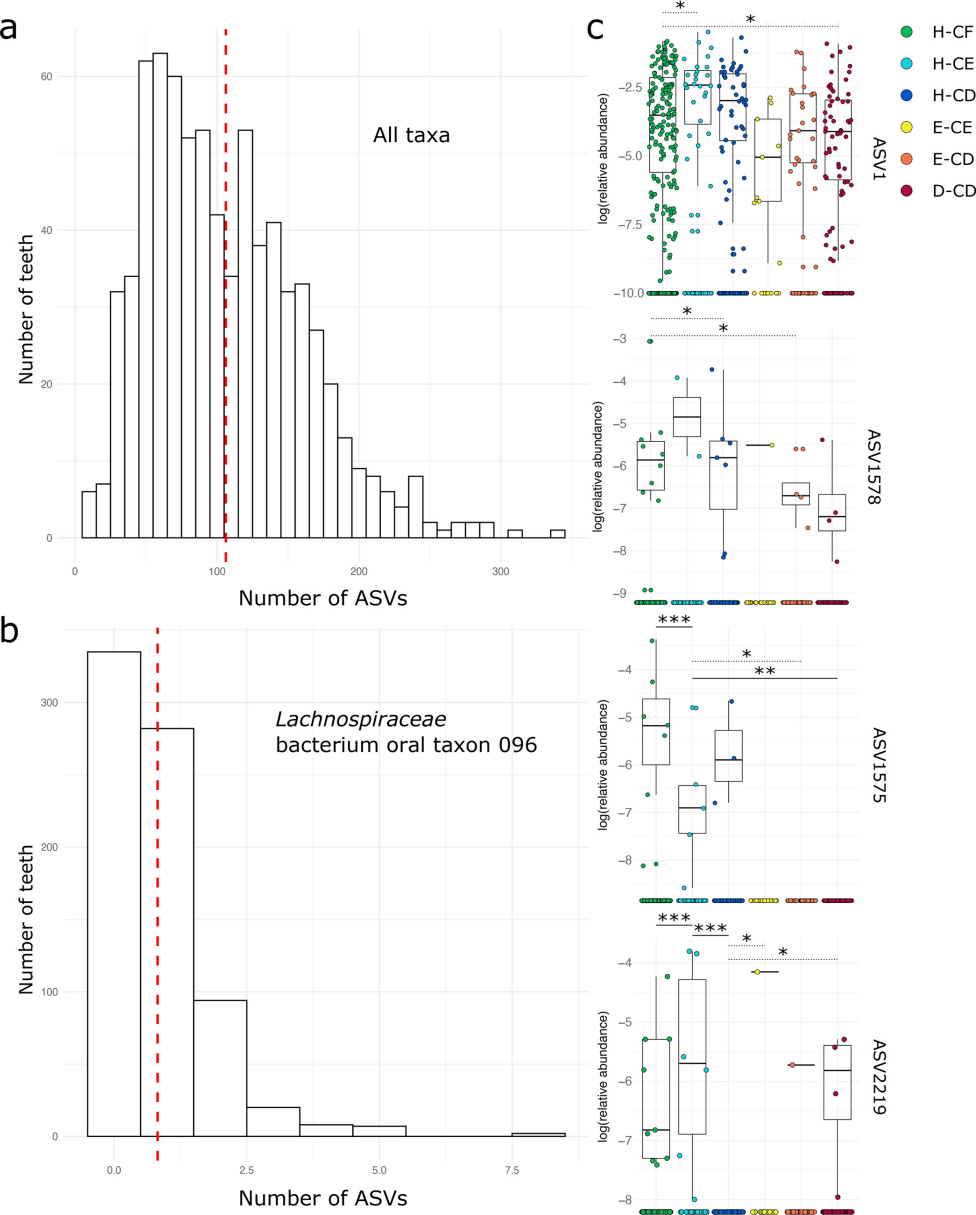
Individual teeth harbor substantial ASV diversity. (a) Histogram of the number of ASVs found on individual teeth. The red dotted line indicates the mean across all teeth. (b) Histogram of the number of ASVs found in a single bacterial species (*Lachnospiraceae* bacterium oral taxon 096). The red dotted line indicates the mean across all teeth. (c) Log-transformed relative abundance box plots of four differentially abundant *Lachnospiraceae* bacterium oral taxon 096 ASVs across tooth health categories. *, *P* ≤ 0.05; **, *P* ≤ 0.01; ***, *P* ≤ 0.001. Comparisons between groups that are not significant after FDR adjustment are indicated by dotted lines.

To visualize microbial intersections at the ASV level between individuals of a particular tooth health or HIV status group, we next generated UpSet plots, which visually represent shared ASVs across groups (Fig. 1c and d). Most ASVs were shared across all individuals in our data set, and few were uniquely found in any one group. Overall, we found that the community in plaque was dominated by *Firmicutes* (median relative abundance, 28%), *Bacteroidetes* (median relative abundance, 23%), *Proteobacteria* (median relative abundance, 20%), *Fusobacteria* (median relative abundance, 13%), *Actinobacteria* (median relative abundance, 7%), and *Spirochaetes* (median relative abundance, 3%) (Fig. S4). The most common genera included Streptococcus (median relative abundance, 36%), *Rothia* (median relative abundance, 32%), *Veillonella* (median relative abundance, 32%), and *Arachnia* (median relative abundance, 30%) (Table S5). Our *rpoC* gene fragment approach identified substantial ASV-level diversity within oral species. On average, nine ASVs (SD, 12) were generated for each species-level taxonomic assignment, with a maximum of 94 ASVs generated for a single species (Campylobacter concisus) (Table S6). The extent of ASV-level diversity is especially pronounced among important groups of oral commensals. For example, we generated 155 ASVs assigned to 20 species of Streptococcus, the most diverse of which was Streptococcus cristatus (39 ASVs). ASVs assigned to any one genus or species have a patchy distribution across children, so no one ASV is clearly dominant, and instead, the bacterial community of a single plaque sample is highly individualistic at the ASV level. Consider, for example, that of the 155 ASVs generated for Streptococcus, 81% were found in fewer than 50 children and the most frequently detected ASV assigned to *S. cristatus* was present in just 12% of all plaque samples. We also detected substantial ASV diversity at the individual-tooth level, though we found no significant differences in community alpha diversity by tooth type (i.e., molar versus incisor) in adult teeth as measured by Shannon diversity (*P* = 0.13). On average, a single tooth harbored 64 distinct species (SD, 25) (Table S7) and 106 total ASVs (SD, 54; maximum, 339) (Fig. 2a), of which 96 (SD, 49) were assigned to the species level. The plaque community of an individual tooth, therefore, often includes multiple distinct ASVs assigned to a single species. For example, of the 13 distinct ASVs assigned to *Lachnospiraceae* bacterium oral taxon 096, a maximum of eight were found on a single tooth (Fig. 2b). The distribution of ASVs belonging to a single species tends to be variable across health groups, with some ASVs increasing or decreasing along the six progressive disease states, a pattern recently documented among Streptococcus spp. (28). For example, while the relative abundance of *Lachnospiraceae* bacterium oral taxon 096 ASV1 and ASV1578 is relatively equivalent across samples from all six tooth health groups, ASV1575 is significantly higher in the H-CF than the H-CE group (Wilcoxon signed-rank test, false discovery rate [FDR] = 0.009) and significantly higher in H-CE compared to D-CD (Wilcoxon signed-rank test, FDR = 0.01). Similarly, *Lachnospiraceae* bacterium oral taxon 096 ASV2219 is significantly more abundant in the H-CE group than both the H-CF (Wilcoxon signed-rank test, FDR = 0.002) and H-CD (Wilcoxon signed-rank test, FDR = 0.0003) groups (Fig. 2c).

Along with known members of the oral cavity, we detected taxa that may be signs of the local environment. For example, four relatively high-frequency ASVs were assigned as Salinivirga cyanobacteriivorans, an anaerobic bacterium originally isolated from hypersaline mats (29, 30). Upon further investigation, the closest match to this ASV using BLAST (31) against the NCBI nucleotide database is another environmental (i.e., not host-associated) taxon, Cytophaga hutchinsonii (CP000383.1), at low query coverage (76%) and low sequence identity (78.14%). ASVs assigned to Salinivirga cyanobacteriivorans were found in 32% of all plaque samples examined here, with an average read count of 506 (± 2,011), and thus may represent a novel bacterial group that is not represented in public databases, though it remains unclear if it is a true oral resident or environmental in origin. It is also possible that these ASVs are contaminants. Importantly, however, only two of the ASVs assigned to Salinivirga cyanobacteriivorans (ASV42 and ASV9) were detected in our blanks and at very low frequency (average read counts, 5 ± 32 and 1 ± 7, respectively); thus, they were unlikely to have been introduced during library preparation.

**FIG 3:**
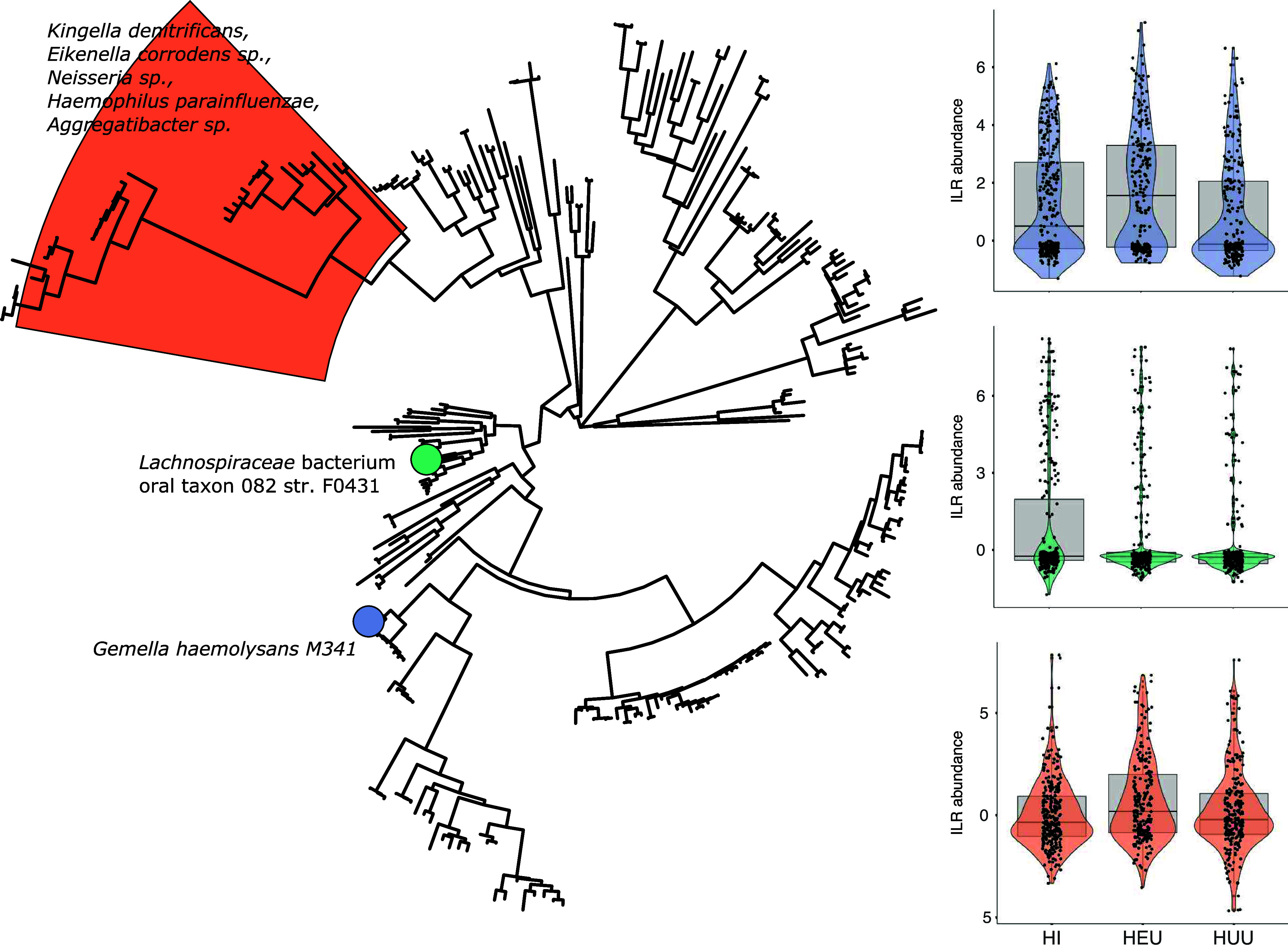
Differential abundance of bacterial taxa enriched in HI, HUU, and HEU individuals. Subsampled phylogenetic tree of ASVs generated in this study with highlighting to show where individual ASVs (circles) or larger phylogenetic groups were identified as major sources of variation between HIV groups. Corresponding violin charts illustrate box plots of abundance after isometric log ratio transformation of raw counts. Colors correspond to highlighted sections of the phylogenetic tree.

We next performed phylofactor analysis to identify ASVs or groups of ASVs that may be predictive of HIV status independent of tooth health and found a single ASV assigned to Gemella haemolysans to be significantly more abundant among plaque collected from HEU children than in plaque collected from HI children (*P* = 0.02) (Fig. 3) and HUU children (*P* < 0.001). The same ASV was also significantly more abundant among HI children than HUU children (*P* = 0.004). We also detected a single ASV assigned as *Lachnospiraceae* bacterium oral taxon 082 strain F0431 that was more abundant in HI children than HUU children (*P* = 0.001) and a large paraphyletic clade of 31 ASVs that were significantly more abundant in HEU children than HI children (*P* = 0.001) and HUU children (*P* = 0.02), consisting of species belonging to the genera *Kingella*, *Eikenella*, *Neisseria*, Haemophilus, and *Aggregatibacter*.

**FIG 4:**
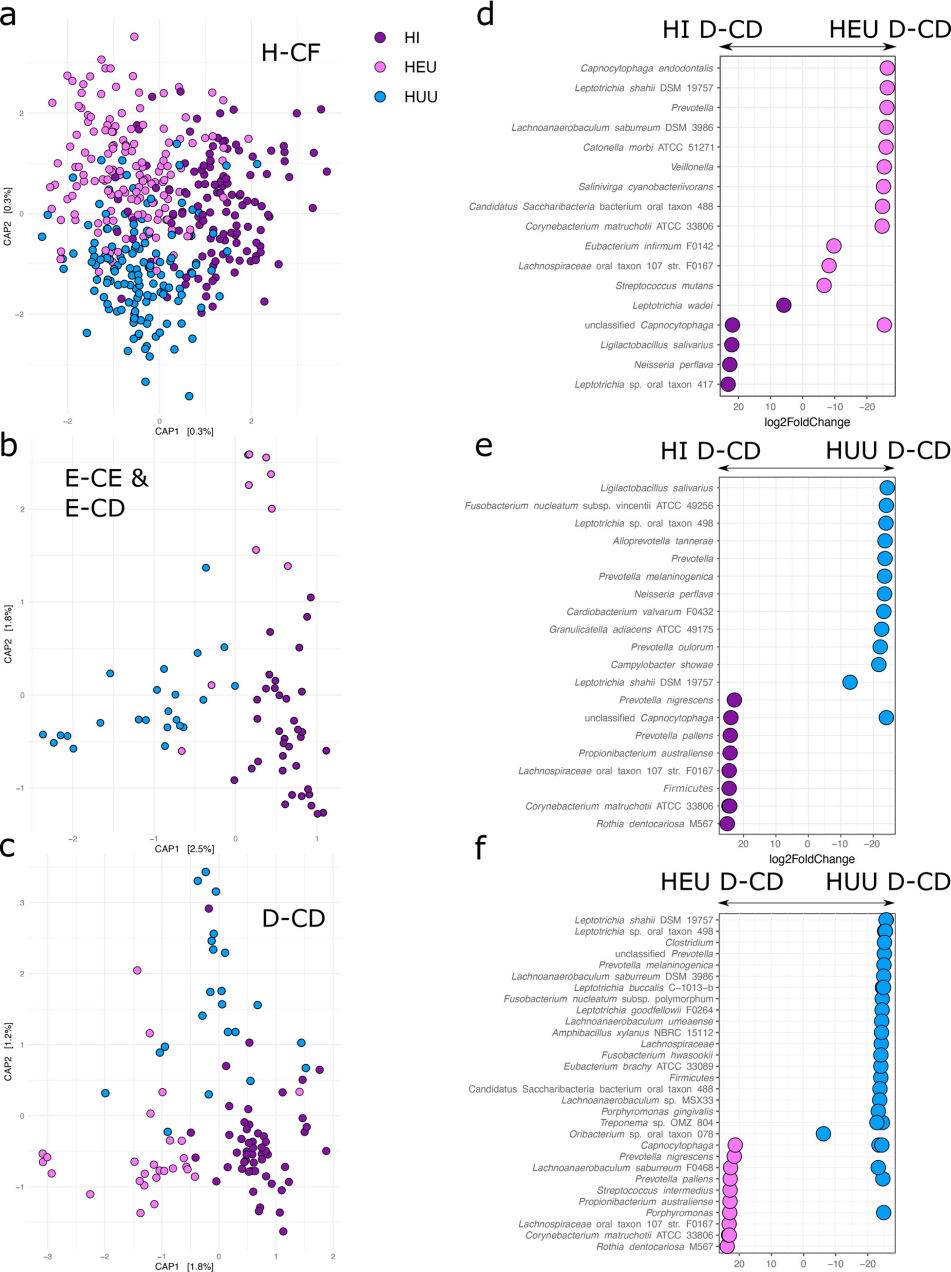
Differences in the microbial community of HI compared to HEU and HUU children are more pronounced in cavitated than noncavitated teeth. (a) Capscale plot of bacterial beta diversity as measured by a Bray-Curtis dissimilarity matrix of H-CF plaque samples, only colored by HIV status. (b) Capscale plot of bacterial beta diversity as measured by a Bray-Curtis dissimilarity matrix of D-CE or E-CE plaque samples, colored by HIV status. (c) Capscale plot of bacterial beta diversity as measured by a Bray-Curtis dissimilarity matrix of D-CD plaque samples only, colored by HIV status. (d) Log fold change of statistically significant taxa differentiating HI and HEU children in D-CD plaque samples. (e) Log fold change of statistically significant taxa differentiating HI and HUU children in D-CD plaque samples. (f) Log fold change of statistically significant taxa differentiating HEU and HUU children in D-CD plaque samples.

Because tooth health is a confounding factor in determining the impact of HIV on the supragingival plaque microbiome, we next investigated the effect of HIV on individual tooth health categories. We found that the impact of HIV was modulated by tooth health, with some categories being more conspicuously impacted than others. For example, the accuracy of predicting HIV status was weak among H-CF plaque samples, with HI correctly predicted in only 53% of cases, HEU in 40% of cases, and HUU in 47% of cases. Conversely, plaque samples collected from D-CD teeth were correctly identified as HI with 80% accuracy, while the correct identification of HEU and HUU remained relatively low (45% and 30%, respectively). Capscale plots of Bray-Curtis dissimilarity matrices document an increased divergence of bacterial communities among HIV status groups as caries progresses (Fig. 4a to c).

In agreement with our random forest classification model and beta diversity metrics, we found the fewest taxa within H-CF plaque samples differentiating HIV groups with DESeq2 analysis. A single ASV assigned to Prevotella oralis was depleted in H-CF plaque samples of children who were HI compared to HUU H-CF (Benjamini-Hochberg procedure *P* ≤ 0.001) and a single ASV assigned to Corynebacterium matruchotii ATCC 33806 was enriched in H-CF plaque samples from children who were HI compared to HEU H-CF samples (BH *P* ≤ 0.001). Two ASVs were enriched in HEU H-CF plaque samples compared to HI H-CF samples (Capnocytophaga gingivalis [BH *P* ≤ 0.001] and *Leptotrichia* sp. oral taxon 417 [BH *P* ≤ 0.001]). We detected no ASVs that were significantly enriched or depleted when comparing HEU H-CF and HUU H-CF plaque samples. This contrasts with the D-CD plaque samples, where more ASVs were found to be differentially abundant between HIV status groups (Fig. 4d to f). ASVs enriched in HI D-CD as opposed to HEU D-CD include Leptotrichia wadei, an unclassified *Capnocytophaga* ASV, Ligilactobacillus salivarius, Neisseria perflava, and *Leptotrichia* sp. oral taxon 417. ASVs enriched in HI D-CD compared to HUU D-CD include Prevotella nigrescens, an unknown *Capnocytophaga* ASV, Prevotella pallens, Propionibacterium australiense, *Lachnospiraceae* oral taxon 107 strain F0167, an unknown species of *Firmicutes*, Corynebacterium matruchotii ATCC 33806, and Rothia dentocariosa M567. The closest match to the ASV assigned to *Firmicutes* was the environmental species Aminipila terrae (32), with a query coverage of 99% and sequence identity of 80.08% compared to the NCBI nucleotide database using BLAST. Like the ASVs assigned to Salinivirga cyanobacteriivorans, this ASV may represent a transient or environmental taxon.

Next, we defined potential core taxa according to HIV status within tooth health groups as those present across all members of a group after center log-ratio transformation using the R microbiome package (33). Core taxa were considered “characteristic” of a group if they were uniquely identified as a core taxon among members of that group and no other (though this does not preclude that taxon from being present among a subset of members of other groups). Characteristic taxa in HI H-CF plaque samples included Eubacterium yurii subsp. *margaretiae* ATCC 43715 (ASV143), Rothia dentocariosa ATCC 17931 (ASV11), Veillonella parvula (ASV43), and *Lachnospiraceae* bacterium oral taxon 082 strain. F0431 (ASV3). Two ASVs assigned to an unknown species of *Fusobacterium* (ASV188) and Haemophilus parainfluenzae (ASV27) were characteristic core taxa among HEU H-CF individuals, and a single ASV assigned to Leptotrichia wadei (ASV10) was a characteristic core taxon among HUU H-CF samples. Among D-CD samples, core taxa characteristic of HI included Campylobacter showae (ASV65), *Leptotrichia* sp. oral taxon 225 strain F0581 (ASV215), and Streptococcus mutans (ASV6). Core ASVs characteristic of HEU D-CD include Eubacterium brachy ATCC 33089 (AV61), Streptococcus mutans (ASV7), Peptostreptococcus stomatis DSM 17678 (ASV100), *Porphyromonas* sp. oral taxon 279 (ASV95), Prevotella marshii DSM 16973 (ASV110), and Streptococcus sanguinis (ASV882). Core taxa characteristic of HUU D-CD include an unknown member of Haemophilus (ASV19), Streptococcus sanguinis SK150 (ASV40), Alloprevotella tannerae ATCC 51259 (ASV157), and Haemophilus parainfluenzae (ASV14).

**FIG 5:**
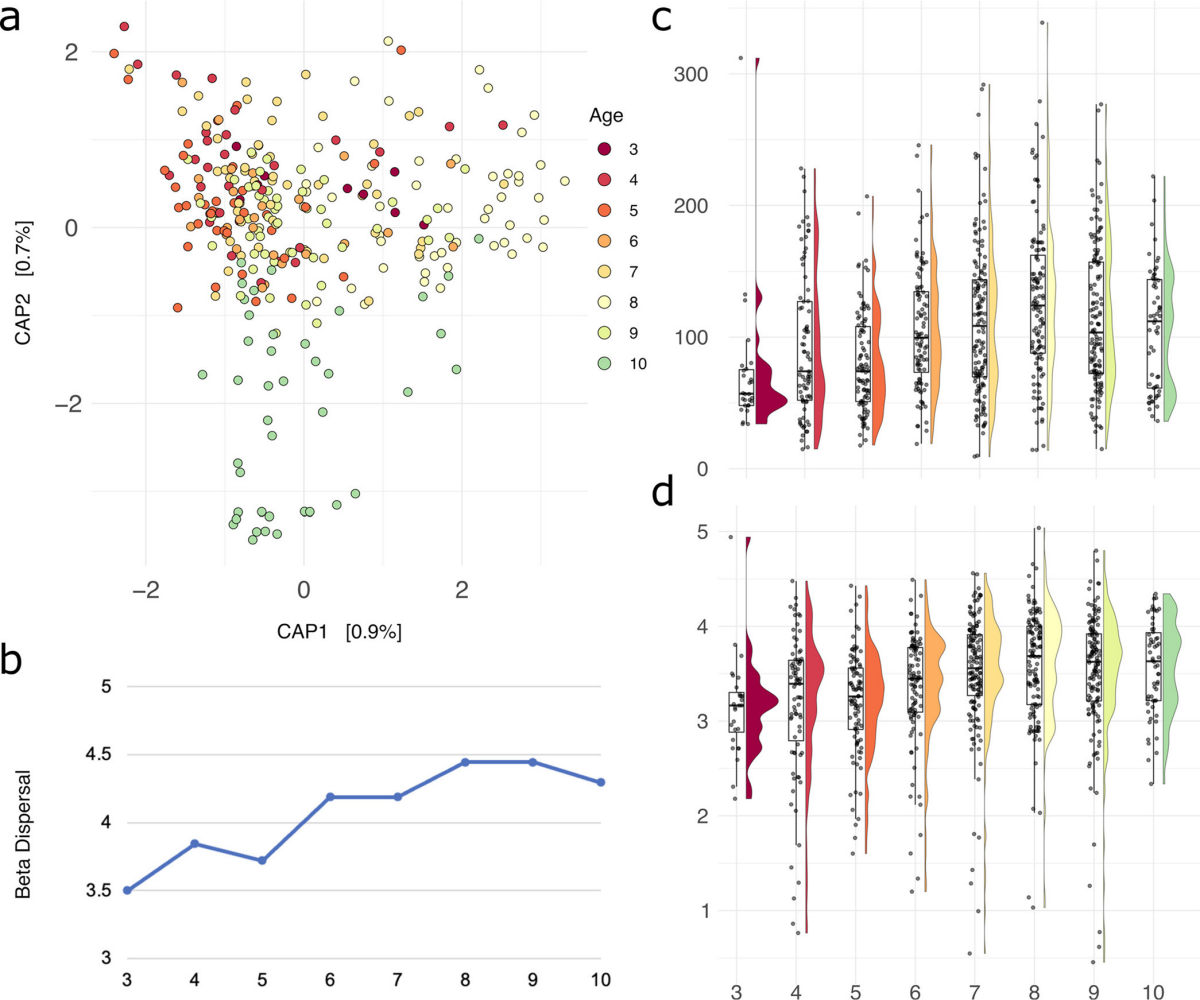
Diversity and dispersal of the oral microbiome increases with age. (a) Capscale plot of bacterial beta diversity in all samples as measured by a Bray-Curtis dissimilarity matrix, colored by age. (b) Increasing beta dispersal of all samples among older children. (c) Observed ASV count by age category in years. (d) Shannon diversity by age category in years.

As there is considerable interest in the impact of long-term antiretroviral therapy (ART) treatment on the oral microbiome and incidence of caries in children living with HIV, we next investigated changes in diversity metrics within tooth health groups by age of the individual. As the number of plaque samples collected from the youngest (3 years old) and oldest (10 years old) were relatively low (Fig. S5), we considered differences only between children 4 and 9 years of age. We found that younger children had lower community richness than older children and that this difference was statistically significant between the oldest children and children 6 years and younger (*P* < 0.04). A capscale plot of beta diversity dissimilarity highlights this distinction, wherein younger children (≤6 years old) exhibit a more cohesive clustering pattern, while patterns in children older than 6 are more disperse (Fig. 5a). Interestingly, beta dispersal also increases in older children (*P* = 0.001) (Fig. 5b). This effect is driven by plaque samples collected from HI children, wherein children 6 years and older have higher alpha diversity (observed ASVs, *P* = 0.003; Shannon, *P* = 0.001) and higher beta dispersal (*P* = 0.001) than HEU and HUU children, where the difference is not statistically significant regardless of tooth health status. Additionally, plaque samples from adult teeth collected from HI children had higher diversity than samples collected from primary teeth as measured by both Shannon diversity (*P* = 0.02) and the total number of observed ASVs (*P* = 0.02) (Fig. 5c and d). There were no statistically significant differences in community richness between primary and adult teeth in HEU or HUU children.

**FIG 6:**
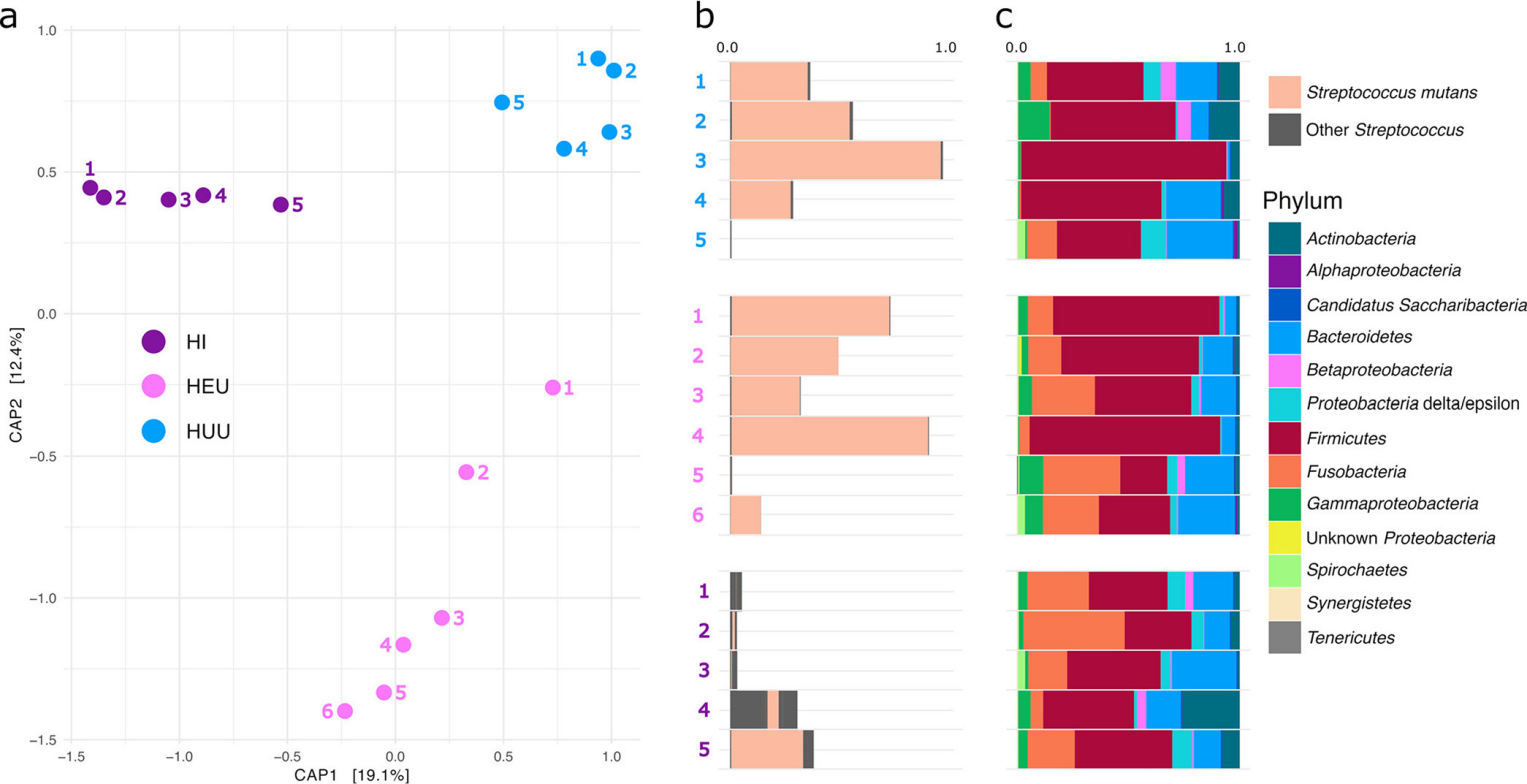
Site-specific intraindividual variation of the oral microbiome and the cariogenic pathogen Streptococcus mutans. (a) Capscale plot of bacterial beta diversity as measured by a Bray-Curtis dissimilarity matrix of 16 individual plaque samples collected from D-CD teeth from three individuals representing one of each of the HIV status groups in this study. Plaque samples from the same individual are colored by HIV status and individual. (b) The relative abundance of S. mutans and other Streptococcus spp. in each plaque sample corresponding to the capscale plot. Number and color link samples to the capscale plot, S. mutans relative abundance, and phylum-level taxonomic bar charts. (c) Phylum-level taxonomic bar charts illustrating changing frequencies of the top phyla among individual plaque samples shown in the capscale plot.

Our single-tooth sampling strategy provides improved resolution of small-scale ecological changes among teeth with the same health status and at the individual level. To illustrate this at the individual level, we analyzed 16 D-CD teeth from three children representing each of the three HIV status groups. We found that differences between individual teeth can be quite stark, and in D-CD teeth, these differences are often driven by the total proportion of Streptococcus mutans in the community (Fig. 6). It is important to note that while S. mutans tends to be a dominant taxon in later-stage caries, it can be found at lower frequency in earlier stages of caries or even in otherwise caries-free teeth (Fig. S6). Moreover, the frequency of S. mutans in plaque samples collected from late-stage caries is individual specific and varies by tooth in the same mouth (Fig. 6b). Interestingly, the abundance of S. mutans ASVs varies across tooth health status, with some ASVs (e.g., ASV12 and ASV6) increasing in abundance according to our six progressive health stages, while others (e.g., ASV63) are more prevalent among healthy teeth (H-CF and H-CE) and teeth with dentin lesions (D-CD) but found at very low frequency in teeth with enamel caries (E) (Fig. S7). As expected, however, Streptococcus mutans ASVs tend to be significantly higher in later stages of caries (E-CD and D-CD) than in other health groups.

**FIG 7:**
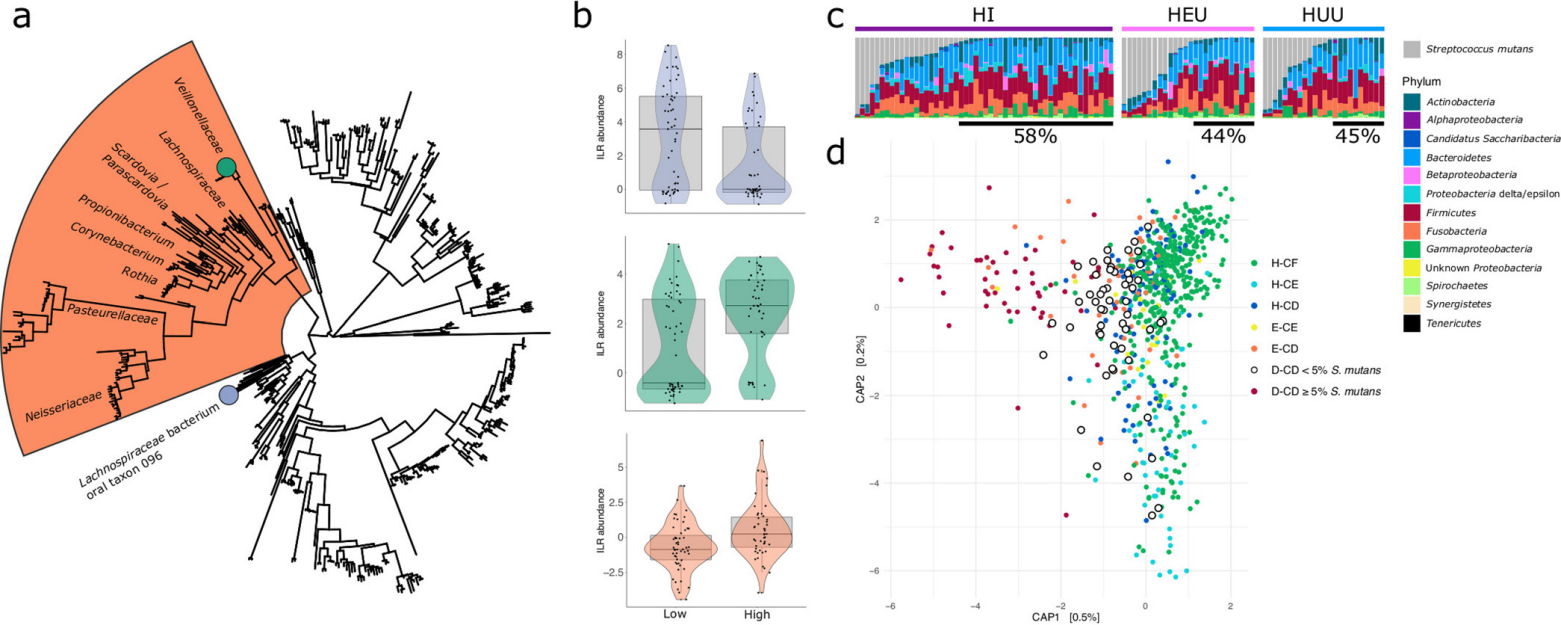
Teeth with severe caries (D-CD) have various abundances of Streptococcus mutans. (a) Subsampled phylogenetic tree of ASVs generated in this study with highlighting to show where individual ASVs (circles) or larger phylogenetic groups were identified as major sources of variation between low- and high-S. mutans D-CD samples. Any ASV assigned to S. mutans was removed before the analysis was performed. (b) Violin plots illustrating the isometric log ratio (ILR)-transformed abundances of differentially abundant taxa corresponding to the phylogenetic tree. (c) Relative abundance of S. mutans compared to other taxa in D-CD teeth, split by HIV status. Black bars under taxonomy bar charts highlight samples with less than 5% S. mutans. (d) Beta diversity of all samples colored by tooth health status. Samples with low S. mutans are highlighted and cluster more closely to healthy teeth.

To investigate possible alternative species driving caries formation or community changes that may explain the low proportion of S. mutans among a subset of D-CD plaque samples, we ran diversity estimates, differential abundance tests, and random forest classification estimates on D-CD plaque samples with less than 5% total abundance of S. mutans. As expected, supragingival plaque samples with a low relative abundance of S. mutans had significantly higher alpha diversity than those with a higher relative abundance of S. mutans, as measured by both the number of ASVs (*P* = 0.02) and Shannon diversity (*P* < 0.001). We also found that D-CD samples with high levels of S. mutans were enriched for a large clade of 135 ASVs primarily assigned to *Veillonellaceae*, *Lachnospiraceae*, *Scardovia* sp., *Parascardovia* sp., *Propionibacterium*, *Corynebacterium*, *Rothia* sp., *Pasteurellaceae*, and *Neisseriaceae* (*P* = 0.002) using phylofactor analysis (Fig. 7a and b). Within *Veillonellaceae*, a single ASV assigned to an unknown member of *Veillonella* was identified as higher abundant among D-CD samples with high levels of Streptococcus mutans (*P* = 0.0003), while a single ASV assigned to *Lachnospiraceae* bacterium oral taxon 096 was found to be more abundant among D-CD samples with relatively low S. mutans (*P* = 0.005). The closest match in the NCBI nucleotide database for the unknown species of *Veillonella* is *V. parvula* strain SKV38 (LR778174.1), with 99.58% sequence identity and 100% query coverage. After removing all S. mutans ASVs, we were able to accurately identify samples with high levels of S. mutans with 77% accuracy and those with low levels of S. mutans with 62% accuracy using random forest analysis (full results and a confusion matrix can be found in Table S8). Bacterial taxa that were important for defining the random forest model included Propionibacterium acidifaciens, Veillonella parvula, and Scardovia wiggsiae which were also detected as differentially abundant taxa in high S. mutans D-CD plaque samples by phylofactor analysis. Interestingly, the frequency of D-CD teeth with low S. mutans was slightly higher among children who were HI (*P* = 0.06) with a mean abundance of S. mutans of 14%, HEU children with a mean abundance of 25%, and HUU children with a mean abundance of 24%. Among D-CD teeth from HI children, 58% have a low proportion of S. mutans (<5% total abundance), 44% of D-CD HEU teeth have low levels of S. mutans, and 45% of D-CD HUU teeth have low levels S. mutans (Fig. 7c). Importantly, D-CD teeth among individuals of all HIV statuses with low levels of S. mutans cluster more closely with healthy teeth than among those with high levels of S. mutans (Fig. 7d).

Finally, along with taxa that are generally identified as cariogenic, we also detected several suspected periodontal pathogens, including three ASVs assigned to Treponema denticola, two ASVs assigned to Porphyromonas gingivalis, and eight ASVs assigned to Tannerella forsythia at overall low frequency (¯, 0.56%, ± 2.38%) in 28% of all plaque samples. The prevalence of these potential periodontal pathogens was not associated with HIV status, nor were these more frequent in children with active caries. In fact, children with relatively good oral health had the highest prevalence of these species, with 30% of H-CF teeth and 35% of H-CE teeth having reads assigned to one or more taxa, the most common of which were P. gingivalis and *T. forsythia*. Full ASV frequency data can be found in Table S9.

## DISCUSSION

To date, most metataxonomic-based surveys of host-associated microbial communities have targeted one or more hypervariable regions of the 16S rRNA gene, with some exceptions (for example, see references 34–36). There are a range of known limitations to 16S rRNA gene fragment amplicon sequencing, including copy number variation across closely related bacterial taxa, high levels of horizontal gene transfer, and poor or incorrect phylogenetic resolution (37–39). As an alternative amplicon approach, we designed primers targeting a fragment of the bacterial *rpoC* gene to survey the oral microbiome. We adopted this approach because recent research has demonstrated the inability of commonly used 16S rRNA amplicon sequencing techniques to resolve species- or strain-level diversity among important oral groups (i.e., Streptococcus spp.) (28) and *in silico* analysis of *rpoC* had previously identified it as a promising alternative with improved phylogenetic resolution to 16S rRNA sequencing for microbial ecology (37). Our approach documents substantial ASV diversity within important oral species and highlights yet-underappreciated inter- and intraindividual variation in the microbial community across tooth health status and HIV exposure groups. Importantly, our sampling approach allowed us to document substantial diversity across teeth in the same mouth which is consistent with diverse plaque microhabitats previously described (40). The results presented here also corroborate prior observations of high genetic diversity among common oral species that is not well captured by 16S rRNA gene-fragment metataxonomics alone (28, 41, 42). Given the functional diversity and pathogenic potential of very closely related bacterial species and strains, a more precise taxonomic interrogation of the plaque microbiome is necessary to understand the role of microbes in the development of caries, particularly in this novel population of children with HIV. For example, in the current study, we detected substantial ASV diversity within Streptococcus sp. Members of Streptococcus have a range of known metabolic behavior (for example, see reference 43); thus, the level of diversity detected here indicates widespread within-species strain diversity with unknown functional outcomes. Alternative marker genes, such as that used in the current study, may provide improved taxonomic resolution but also highlight our limited understanding of the full diversity of the oral microbiome. Consider, for example, that multiple ASVs that were assigned as *Lachnospiraceae* bacterium oral taxa were found at high frequency among many plaque samples and were differentially abundant between groups. While *Lachnospiraceae* bacteria are known members of the human digestive tract (44), little is known about their functional role in the oral cavity. Furthermore, we detected ASVs that were highly abundant across samples but had no close match in our database and could represent bacteria originating from the local environment. Distinguishing bacteria that are true members of the plaque community from those that may be transient is an unresolved complication in studies of host-associated microbial ecology that may be improved by a more comprehensive taxonomic survey of the microbiome.

Confirming similar patterns in previous studies, we found that the community composition of the supragingival plaque microbiome of children living with HIV is distinct from that of the microbiome of children who had been exposed to or had never been exposed to the virus, though we did not detect significant differences in community richness (45, 46). Moreover, these differences are more evident in later-stage caries development. Plaque samples from healthy teeth tend to have more similar microbial communities, regardless of overall oral health or HIV status. Plaque samples in late-stage caries, however, are more divergent among the three HIV groups. This is important, as it suggests that the influence of HIV on caries progression is more severe as the disease progresses, which may partially explain the higher prevalence of severe caries observed in this cohort. Contrary to our expectations, the increased incidence and severity of caries in children living with HIV does not appear to be driven by an increase in acidogenic bacteria, and instead, we found large-scale phylogenetic depletion of specific taxa thought to be normal commensals of the oral cavity (e.g., Haemophilus parainfluenzae, Kingella denitrificans, and Eikenella corrodens) as well as those associated with caries development (e.g., Streptococcus mutans, Veillonella parvula, and Scardovia wiggsiae). Interestingly, D-CD plaque samples collected from children living with HIV tended to have a lower mean frequency of the cariogenic bacterium Streptococcus mutans than samples from other groups with severe caries, though this effect did not reach statistical significance (*P* = 0.06). This is in contrast to our initial expectations but was observed previously when children living with HIV were compared to exposed but uninfected children (46). These results suggest that the supragingival plaque community in HI D-CD samples is characterized by larger ecological disruptions and not simply the proliferation of specific cariogenic bacteria. It may be the case that HIV infection, the continued use of ART, or their combination affects the relative stability of the oral microbiome over time, resulting in a more volatile community structure affecting both cariogenic and commensal members of the oral microbiome, though further longitudinal analyses are needed.

Importantly, Streptococcus mutans was also not substantially higher in teeth with enamel lesions, independent of HIV status, which supports previous site-specific studies that have found that S. mutans is not an early instigator of caries development (47). Instead, our results confirm that while S. mutans may be a dominant taxon in late-stage caries, the frequency can vary considerably from tooth to tooth, even in the same mouth (48), and the plaque community of a tooth with an active dentin cavity with low abundance of S. mutans is more similar to the community found on a healthy tooth. Because plaque samples collected at a single time point represent only a brief ecological snapshot of the oral community, it is possible that the frequency of S. mutans on a single tooth is a function of a shifting microbial ecology during caries intensification wherein the population of S. mutans rapidly expands, lowering the surrounding pH until only it and other acidophilic bacteria can thrive, followed by ecological collapse of the microbial community, which is then repopulated. While D-CD plaques collected from children living with HIV have, in general, a lower frequency of S. mutans, D-CD teeth with high S. mutans concurrently had a high abundance of other known or suspected cariogenic taxa, including Propionibacterium acidifaciens, Veillonella parvula, and Scardovia wiggsiae (49–53), independent of HIV status, which attests to the multimicrobial nature of caries development.

Finally, we documented an increase in bacterial diversity but a decrease in community cohesion in older children living with HIV, so that the microbial community becomes more individualistic over time. Age-related changes to the oral microbiome in children have been observed (45, 54–58) and are likely partially driven by structural modifications to the oral cavity during replacement of the primary dentition or hormonal changes (56). Moreover, the oral microbiome is relatively unstable (59) and is more directly impacted by the individual’s environment than other host-associated microbiomes (60). Higher dispersal of variation across individuals or individual teeth in older children living with HIV than in HEU or HUU children suggests that this instability is exacerbated in the context of HIV infection and/or its treatment. Further longitudinal studies of children living with HIV are needed to better understand the impact of these age-related changes in the progression of tooth decay and other oral diseases.

The study presented here is the first to examine the impact of HIV infection and exposure on the supragingival plaque microbiome as it relates to caries development in a sub-Saharan African population. Despite increased availability and use of ART, the global burden of HIV remains especially high in sub-Saharan Africa, and Nigeria has the second highest prevalence of HIV globally, affecting approximately 1.9 million people, 35% of whom are children (45, 61, 62). Given the comorbidity of HIV and oral diseases, including tooth decay, a better understanding of the diversity and mechanisms driving microbial community dynamics may provide targeted avenues of intervention for caries progression in children living with HIV.

### Conclusions.

Our results document the complexity of caries development in children with HIV from a microbial perspective and illustrate the need for a more detailed taxonomic and functional profile of the oral microbiome in the context of caries and HIV infection. In agreement with previous research investigating the role of microbial species in the etiology of tooth decay (for example, see references 53 and 63), our data support the notion that while Streptococcus mutans is an important contributor to late-stage caries intensification, it does not appear to be a major influence in the initiation of cariogenesis, and instead, extensive and increasingly individualistic ecological changes in the plaque biofilm are responsible for the increased frequency of caries observed in children living with HIV.

## MATERIALS AND METHODS

Informed consent was obtained from all parents, guardians, or caregivers, and children 8 years and older provided assent before joining the study—University of Maryland Baltimore (HP-00084081), Rutgers State University of New Jersey (Pro2019002047), and University of Benin Teaching Hospital, Benin City (ADM/E22/A/VOL. VII/14713).

Initial sample collection and classification are as described in reference 24. Briefly, supragingival plaques were collected from teeth using a sterile curette and placed into a sterile 2-mL cryogenic vial containing 500 μL of RNAlater (Invitrogen, Carlsbad, CA). Collected plaque samples were placed immediately on ice and stored at −80°C within 2 h of collection. Samples with poor PCR amplification or low read depth postsequencing were not included in downstream analyses, leaving 748 high-quality plaque samples from 484 children ranging in age from 3 to 10 years old. A total of 295 plaque samples were collected from children living with HIV (HI), 224 from children who were exposed to HIV perinatally but tested negative for the disease (HEU), and 230 from children with no exposure to HIV (HUU).

We extracted DNA from each sample using the DNeasy PowerBiofilm kit (Qiagen, Valencia, CA, USA) following the manufacturer’s protocol. DNA yield postextraction was assessed using a Qubit fluorometer (Invitrogen, Carlsbad, CA). Extraction blanks were generated for each round of extraction using molecular-grade water to trace sources of external contamination.

We amplified each sample using custom Illumina adapters with the addition of primers targeting an approximately 478-bp sequence of the bacterial *rpoC* gene: rpoCF (5′-MAYGARAARMGNATGYTNCARGA-3′) and rpoCR (5′-GMCATYTGRTCNCCRTCRAA-3′) (Table S10). Each PCR consisted of 0.5 μL each of the forward and reverse primers, 10 μL of water, 4 μL of template DNA, and 10 μL of Platinum Hot Start PCR master mix (2×) (Invitrogen, Carlsbad, CA) for a total of 25 μL per reaction. Thermocycler conditions were as follows: 94°C for 3 min followed by 41 cycles of 94°C for 45 s, 39.5°C for 1 min, and 72°C for 1 min 30 s. A final elongation step was performed for 10 min at 72°C. Sufficient amplification was confirmed both by gel electrophoresis and with a Qubit fluorometer (Invitrogen, Carlsbad, CA). PCR blanks (molecular-grade water) were amplified and sequenced in parallel to all samples. Samples were pooled at equimolar concentrations before sequencing using V3 2 × 300 paired-end sequencing chemistry on the Illumina MiSeq platform. Mock communities of six common oral bacteria (ATCC MSA-1004) and two pure-culture bacterial taxa (Escherichia coli and Staphylococcus aureus) were sequenced in triplicate in a manner identical to true samples as positive controls. Results for the mock community can be found in Fig. S8.

First, we trimmed primer and adapter sequences from the demultiplexed samples using Cutadapt v.2.8 (64). We then quality-filtered, merged, removed chimeric sequences, and generated ASVs using DADA2 v.1.22 (65). Samples with fewer than 4,000 reads after quality filtering and merged amplicons shorter than 450 bp were removed from downstream analyses. Given the high degeneracy of our primers, we next filtered ASVs with a prevalence frequency threshold of 1%, so that any ASV not found in at least eight plaque samples was not included in downstream analyses, to minimize the impact of low-frequency ASVs or those that may be the product of priming mismatches. Rarefaction curves of quality-filtered data can be found in Fig. S9. Next, we assigned a predicted taxonomy to each ASV using a custom *rpoC* gene database. Briefly, we generated our custom database by first downloading all complete genomes from NCBI with annotated coding sequences, extracted all annotated *rpoC* genes, and generated a custom Kraken2 database from those sequences. We assigned taxonomy to each ASV using our custom database and Kraken2 v2.1.2 (66) with a confidence score threshold of 0.01.

Downstream data analysis was primarily done within the R version 4.1.0 environment (67). We performed diversity analyses using the libraries PhILR (68), phyloseq (69), and microbiome (33). To account for differences in sequencing depth across samples, PERMANOVA and beta dispersion tests were performed with vegan (70) on PhILR-normalized beta diversity metrics. To visualize differences in beta diversity across samples, we generated capscale plots using a distance-based redundancy analysis approach (27). We next tested the predictive power of sample metadata categories on the microbial community using a random forest classification model with the randomForest (71) and rfUtilities (72) libraries. Finally, differential abundance of specific ASVs and phylogenetic groups of bacteria were calculated with phylofactor (73) and DESeq2 (74).

### Data availability.

The data sets generated and analyzed during the current study are available in the European Nucleotide Archive repository under accession number PRJEB60354. The Conda environment for analytical reproducibility and all bioinformatic scripts used to generate statistics and figures can be found as Jupyter Notebooks at https://github.com/aemann01/domhain/tree/main/2022-HIV_oral_microbiome and are archived on Zenodo under doi:10.5281/zenodo.6815616 (https://doi.org/10.5281/zenodo.6815616).

## References

[1] Global Burden of Disease Collaborative Network. 2020. Global Burden of Disease Study 2019 (GBD 2019). https://vizhub.healthdata.org/gbd-results. Retrieved 1 June 2022.

[2] Casamassimo PS, Thikkurissy S, Edelstein BL, Maiorini E. 2009. Beyond the dmft: the human and economic cost of early childhood caries. J Am Dent Assoc 140:650–657. doi:10.14219/jada.archive.2009.0250. 19491160

[3] Jackson SL, Vann WF, Kotch JB, Pahel BT, Lee JY. 2011. Impact of poor oral health on children’s school attendance and performance. Am J Public Health 101:1900–1906. doi:10.2105/AJPH.2010.200915. 21330579 PMC3222359

[4] Giacaman R. 2018. Sugars and beyond. The role of sugars and the other nutrients and their potential impact on caries. Oral Dis 24:1185–1197. doi:10.1111/odi.12778. 28898520

[5] van Houte J, Lopman J, Kent R. 1996. The final pH of bacteria comprising the predominant flora on sound and carious human root and enamel surfaces. J Dent Res 75:1008–1014. doi:10.1177/00220345960750040201. 8708129

[6] Sansone C, Van Houte J, Joshipura K, Kent R, Margolis HC. 1993. The association of mutans streptococci and non-mutans streptococci capable of acidogenesis at a low pH with dental caries on enamel and root surfaces. J Dent Res 72:508–516. doi:10.1177/00220345930720020701. 8423248

[7] van Houte J, Sansone C, Joshipura K, Kent R. 1991. Mutans streptococci and non-mutans streptococci acidogenic at low pH, and in vitro acidogenic potential of dental plaque in two different areas of the human dentition. J Dent Res 70:1503–1507. doi:10.1177/00220345910700120601. 1774381

[8] van Ruyven FOJ, Lingström P, van Houte J, Kent R. 2000. Relationship among mutans streptococci, “low-pH” bacteria, and iodophilic polysaccharide-producing bacteria in dental plaque and early enamel caries in humans. J Dent Res 79:778–784. doi:10.1177/00220345000790021201. 10728980

[9] Cassone A, Cauda R. 2012. Candida and candidiasis in HIV-infected patients: where commensalism, opportunistic behavior and frank pathogenicity lose their borders. AIDS 26:1457–1472. doi:10.1097/QAD.0b013e3283536ba8. 22472853

[10] Hamza OJ, Matee MI, Simon EN, Kikwilu E, Moshi MJ, Mugusi F, Mikx FH, Verweij PE, van der Ven AJ. 2006. Oral manifestations of HIV infection in children and adults receiving highly active anti-retroviral therapy [HAART] in Dar es Salaam, Tanzania. BMC Oral Health 6:12. doi:10.1186/1472-6831-6-12. 16916469 PMC1559688

[11] Nittayananta W, Talungchit S, Jaruratanasirikul S, Silpapojakul K, Chayakul P, Nilmanat A, Pruphetkaew N. 2010. Effects of long-term use of HAART on oral health status of HIV-infected subjects. J Oral Pathol Med 39:397–406. doi:10.1111/j.1600-0714.2009.00875.x. 20202089 PMC3217232

[12] Coker M, El-Kamary S, Enwonwu C, Blattner W, Langenberg P, Mongodin E, Akhigbe P, Obuekwe O, Omoigberale A, Charurat M. 2018. Perinatal HIV infection and exposure and their association with dental caries in Nigerian children. Pediatr Infect Dis J 37:59–65. doi:10.1097/INF.0000000000001702. 28746260 PMC5725234

[13] Oliveira CAGR, Tannure PN, de Souza IPR, Maia LC, Portela MB, Castro GdA. 2015. Is dental caries experience increased in HIV-infected children and adolescents? A meta-analysis. Acta Odontol Scand 73:481–487. doi:10.3109/00016357.2014.958874. 25765439

[14] Rajonson N, Meless D, Ba B, Faye M, Diby J-S, N'zore S, Datté S, Diecket L, N'Diaye C, Aka EA, Kouakou K, Ba A, Ekouévi DK, Dabis F, Shiboski C, Arrivé E. 2017. High prevalence of dental caries among HIV-infected children in West Africa compared to uninfected siblings. J Public Health Dent 77:234–243. doi:10.1111/jphd.12203. 28233316

[15] Rosier BT, Marsh PD, Mira A. 2018. Resilience of the oral microbiota in health: mechanisms that prevent dysbiosis. J Dent Res 97:371–380. doi:10.1177/0022034517742139. 29195050

[16] Sultan AS, Kong EF, Rizk AM, Jabra-Rizk MA. 2018. The oral microbiome: a lesson in coexistence. PLoS Pathog 14:e1006719. doi:10.1371/journal.ppat.1006719. 29370304 PMC5784999

[17] Evans C, Jones CE, Prendergast AJ. 2016. HIV-exposed, uninfected infants: new global challenges in the era of paediatric HIV elimination. Lancet Infect Dis 16:e92–e107. doi:10.1016/S1473-3099(16)00055-4. 27049574

[18] Jumare J, Datong P, Osawe S, Okolo F, Mohammed S, Inyang B, Abimiku A, The Infant Study Team. 2019. Compromised growth among HIV-exposed uninfected compared with unexposed children in Nigeria. Pediatr Infect Dis J 38:280–286. doi:10.1097/INF.0000000000002238. 30418356

[19] McNally LM, Jeena PM, Gajee K, Thula SA, Sturm AW, Cassol S, Tomkins AM, Coovadia HM, Goldblatt D. 2007. Effect of age, polymicrobial disease, and maternal HIV status on treatment response and cause of severe pneumonia in South African children: a prospective descriptive study. Lancet 369:1440–1451. doi:10.1016/S0140-6736(07)60670-9. 17467514

[20] Nozyce ML, Huo Y, Williams PL, Kapetanovic S, Hazra R, Nichols S, Hunter S, Smith R, Seage GR, Sirois PA, Pediatric HIVAIDS Cohort Study. 2014. Safety of in utero and neonatal ARV exposure: cognitive and academic outcomes in HIV-exposed, uninfected children age 5–13 years. Pediatr Infect Dis J 33:1128–1133. doi:10.1097/INF.0000000000000410. 25361407 PMC4217087

[21] Otieno RO, Ouma C, Ong'echa JM, Keller CC, Were T, Waindi EN, Michaels MG, Day RD, Vulule JM, Perkins DJ. 2006. Increased severe anemia in HIV-1-exposed and HIV-1-positive infants and children during acute malaria. AIDS 20:275–280. doi:10.1097/01.aids.0000200533.56490.b7. 16511422

[22] Le Roux SM, Abrams EJ, Donald KA, Brittain K, Phillips TK, Zerbe A, Le Roux DM, Kroon M, Myer L. 2020. Infectious morbidity of breastfed, HIV-exposed uninfected infants under conditions of universal antiretroviral therapy in South Africa: a prospective cohort study. Lancet Child Adolesc Health 4:220–231. doi:10.1016/S2352-4642(19)30375-X. 31932246 PMC7235356

[23] Shapiro RL, Lockman S, Kim S, Smeaton L, Rahkola JT, Thior I, Wester C, Moffat C, Arimi P, Ndase P, Asmelash A, Stevens L, Montano M, Makhema J, Essex M, Janoff EN. 2007. Infant morbidity, mortality, and breast milk immunologic profiles among breast-feeding HIV-infected and HIV-uninfected women in Botswana. J Infect Dis 196:562–569. doi:10.1086/519847. 17624842

[24] Coker MO, Akhigbe P, Osagie E, Idemudia NL, Igedegbe O, Chukwumah N, Adebiyi R, Mann AE, O'Connell LM, Obuekwe O, Omoigberale A, Charurat ME, Richards VP. 2021. Dental caries and its association with the oral microbiomes and HIV in young children—Nigeria (DOMHaIN): a cohort study. BMC Oral Health 21:620. doi:10.1186/s12903-021-01944-y. 34863179 PMC8642767

[25] Gugnani N, Pandit I, Srivastava N, Gupta M, Sharma M. 2011. International caries detection and assessment system (ICDAS): a new concept. Int J Clin Pediatr Dent 4:93–100. doi:10.5005/jp-journals-10005-1089. 27672245 PMC5030492

[26] Nascimento MM, Liu Y, Kalra R, Perry S, Adewumi A, Xu X, Primosch RE, Burne RA. 2013. Oral arginine metabolism may decrease the risk for dental caries in children. J Dent Res 92:604–608. doi:10.1177/0022034513487907. 23640952 PMC3684231

[27] Legendre P, Anderson MJ. 1999. Distance-based redundancy analysis: testing multispecies responses in multifactorial ecological experiments. Ecol Monogr 69:1–24. doi:10.1890/0012-9615(1999)069[0001:DBRATM]2.0.CO;2.

[28] O'Connell LM, Blouin T, Soule A, Burne RA, Nascimento MM, Richards VP. 2022. Optimization and evaluation of the 30S-S11 rRNA gene for taxonomic profiling of oral streptococci. Appl Environ Microbiol 88:e00453-22. doi:10.1128/aem.00453-22. 35730938 PMC9275224

[29] Ben Hania W, Joseph M, Bunk B, Spröer C, Klenk H-P, Fardeau M-L, Spring S. 2017. Characterization of the first cultured representative of a Bacteroidetes clade specialized on the scavenging of cyanobacteria. Environ Microbiol 19:1134–1148. doi:10.1111/1462-2920.13639. 27943642

[30] Fardeau M-L, Spring S. 2021. Salinivirga, p 1–7. *In* Whitman WB (ed), Bergey’s manual of systematics of Archaea and Bacteria. John Wiley & Sons, Ltd., Hoboken, NJ.

[31] Altschul SF, Gish W, Miller W, Myers EW, Lipman DJ. 1990. Basic local alignment search tool. J Mol Biol 215:403–410. doi:10.1016/S0022-2836(05)80360-2. 2231712

[32] Kim YB, Kim JY, Kim J, Song HS, Whon TW, Lee SH, Yoo S, Myoung J, Son H-S, Roh SW. 2021. Aminipila terrae sp. nov., a strictly anaerobic bacterium isolated from river sediment. Arch Microbiol 203:3163–3169. doi:10.1007/s00203-021-02301-x. 33821299

[33] Lahti L, Shetty S. 2012. microbiome R package.

[34] Ogier J-C, Pagès S, Galan M, Barret M, Gaudriault S. 2019. rpoB, a promising marker for analyzing the diversity of bacterial communities by amplicon sequencing. BMC Microbiol 19:171. doi:10.1186/s12866-019-1546-z. 31357928 PMC6664775

[35] Poirier S, Rué O, Peguilhan R, Coeuret G, Zagorec M, Champomier-Vergès M-C, Loux V, Chaillou S. 2018. Deciphering intra-species bacterial diversity of meat and seafood spoilage microbiota using gyrB amplicon sequencing: a comparative analysis with 16S rDNA V3-V4 amplicon sequencing. PLoS One 13:e0204629. doi:10.1371/journal.pone.0204629. 30252901 PMC6155546

[36] Roux S, Enault F, Bronner G, Debroas D. 2011. Comparison of 16S rRNA and protein-coding genes as molecular markers for assessing microbial diversity (Bacteria and Archaea) in ecosystems. FEMS Microbiol Ecol 78:617–628. doi:10.1111/j.1574-6941.2011.01190.x. 22066608

[37] Hassler HB, Probert B, Moore C, Lawson E, Jackson RW, Russell BT, Richards VP. 2022. Phylogenies of the 16S rRNA gene and its hypervariable regions lack concordance with core genome phylogenies. Microbiome 10:104. doi:10.1186/s40168-022-01295-y. 35799218 PMC9264627

[38] Johnson JS, Spakowicz DJ, Hong B-Y, Petersen LM, Demkowicz P, Chen L, Leopold SR, Hanson BM, Agresta HO, Gerstein M, Sodergren E, Weinstock GM. 2019. Evaluation of 16S rRNA gene sequencing for species and strain-level microbiome analysis. 1. Nat Commun 10:5029. doi:10.1038/s41467-019-13036-1. 31695033 PMC6834636

[39] Louca S, Doebeli M, Parfrey LW. 2018. Correcting for 16S rRNA gene copy numbers in microbiome surveys remains an unsolved problem. Microbiome 6:41. doi:10.1186/s40168-018-0420-9. 29482646 PMC5828423

[40] Morillo-Lopez V, Sjaarda A, Islam I, Borisy GG, Welch JM. 2022. Corncob structures in dental plaque reveal microhabitat taxon specificity. Microbiome 10:145. doi:10.1186/s40168-022-01323-x. 36064650 PMC9446765

[41] Bennett JS, Watkins ER, Jolley KA, Harrison OB, Maiden MCJ. 2014. Identifying Neisseria species by use of the 50S ribosomal protein L6 (rplF) gene. J Clin Microbiol 52:1375–1381. doi:10.1128/JCM.03529-13. 24523465 PMC3993661

[42] Mukherjee C, Beall CJ, Griffen AL, Leys EJ. 2018. High-resolution ISR amplicon sequencing reveals personalized oral microbiome. Microbiome 6:153. doi:10.1186/s40168-018-0535-z. 30185233 PMC6126016

[43] Velsko IM, Chakraborty B, Nascimento MM, Burne RA, Richards VP. 2018. Species designations belie phenotypic and genotypic heterogeneity in oral streptococci. mSystems 3:e00158-18. doi:10.1128/mSystems.00158-18. PMC6299155 30574560

[44] Meehan CJ, Beiko RG. 2014. A phylogenomic view of ecological specialization in the Lachnospiraceae, a family of digestive tract-associated bacteria. Genome Biol Evol 6:703–713. doi:10.1093/gbe/evu050. 24625961 PMC3971600

[45] Coker MO, Mongodin EF, El-Kamary SS, Akhigbe P, Obuekwe O, Omoigberale A, Langenberg P, Enwonwu C, Hittle L, Blattner WA, Charurat M. 2020. Immune status, and not HIV infection or exposure, drives the development of the oral microbiota. Sci Rep 10:10830. doi:10.1038/s41598-020-67487-4. 32616727 PMC7331591

[46] Starr JR, Huang Y, Lee KH, Murphy CM, Moscicki A-B, Shiboski CH, Ryder MI, Yao T-J, Faller LL, Van Dyke RB, Paster BJ, Pediatric HIV/AIDS Cohort Study. 2018. Oral microbiota in youth with perinatally acquired HIV infection. Microbiome 6:100. doi:10.1186/s40168-018-0484-6. 29855347 PMC5984365

[47] Richards VP, Alvarez AJ, Luce AR, Bedenbaugh M, Mitchell ML, Burne RA, Nascimento MM. 2017. Microbiomes of site-specific dental plaques from children with different caries status. Infect Immun 85:e00106-17. doi:10.1128/IAI.00106-17. 28507066 PMC5520424

[48] Gross EL, Beall CJ, Kutsch SR, Firestone ND, Leys EJ, Griffen AL. 2012. Beyond Streptococcus mutans: dental caries onset linked to multiple species by 16S rRNA community analysis. PLoS One 7:e47722. doi:10.1371/journal.pone.0047722. 23091642 PMC3472979

[49] Kameda M, Abiko Y, Washio J, Tanner ACR, Kressirer CA, Mizoguchi I, Takahashi N. 2020. Sugar metabolism of Scardovia wiggsiae, a novel caries-associated bacterium. Front Microbiol 11. doi:10.3389/fmicb.2020.00479. PMC7109253 32269556

[50] Liu S, Chen M, Wang Y, Zhou X, Peng X, Ren B, Li M, Cheng L. 2020. Effect of Veillonella parvula on the physiological activity of Streptococcus mutans. Arch Oral Biol 109:104578. doi:10.1016/j.archoralbio.2019.104578. 31589997

[51] Obata J, Fujishima K, Nagata E, Oho T. 2019. Pathogenic mechanisms of cariogenic Propionibacterium acidifaciens. Arch Oral Biol 105:46–51. doi:10.1016/j.archoralbio.2019.06.005. 31254840

[52] Silva-Boghossian C, Castro GF, Teles RP, De Souza IPR, Colombo APV. 2008. Salivary microbiota of HIV-positive children and its correlation with HIV status, oral diseases, and total secretory IgA. Int J Paediatr Dent 18:205–216. doi:10.1111/j.1365-263X.2007.00864.x. 18384349

[53] Tanner ACR, Mathney JMJ, Kent RL, Chalmers NI, Hughes CV, Loo CY, Pradhan N, Kanasi E, Hwang J, Dahlan MA, Papadopolou E, Dewhirst FE. 2011. Cultivable anaerobic microbiota of severe early childhood caries. J Clin Microbiol 49:1464–1474. doi:10.1128/JCM.02427-10. 21289150 PMC3122858

[54] Crielaard W, Zaura E, Schuller AA, Huse SM, Montijn RC, Keijser BJ. 2011. Exploring the oral microbiota of children at various developmental stages of their dentition in the relation to their oral health. BMC Med Genomics 4:22. doi:10.1186/1755-8794-4-22. 21371338 PMC3058002

[55] Dashper SG, Mitchell HL, Lê Cao K-A, Carpenter L, Gussy MG, Calache H, Gladman SL, Bulach DM, Hoffmann B, Catmull DV, Pruilh S, Johnson S, Gibbs L, Amezdroz E, Bhatnagar U, Seemann T, Mnatzaganian G, Manton DJ, Reynolds EC. 2019. Temporal development of the oral microbiome and prediction of early childhood caries. Sci Rep 9:19732. doi:10.1038/s41598-019-56233-0. 31874981 PMC6930300

[56] Goldberg BE, Mongodin EF, Jones CE, Chung M, Fraser CM, Tate A, Zeichner SL. 2015. The oral bacterial communities of children with well-controlled HIV infection and without HIV infection. PLoS One 10:e0131615. doi:10.1371/journal.pone.0131615. 26146997 PMC4492946

[57] Holgerson PL, Öhman C, Rönnlund A, Johansson I. 2015. Maturation of oral nicrobiota in children with or without dental caries. PLoS One 10:e0128534. doi:10.1371/journal.pone.0128534. 26020247 PMC4447273

[58] Ooshima T, Nishiyama N, Hou B, Tamura K, Amano A, Kusumoto A, Kimura S. 2003. Occurrence of periodontal bacteria in healthy children: a 2-year longitudinal study. Community Dent Oral Epidemiol 31:417–425. doi:10.1046/j.1600-0528.2003.00112.x. 14986909

[59] Ding T, Schloss PD. 2014. Dynamics and associations of microbial community types across the human body. Nature 509:357–360. doi:10.1038/nature13178. 24739969 PMC4139711

[60] Stahringer SS, Clemente JC, Corley RP, Hewitt J, Knights D, Walters WA, Knight R, Krauter KS. 2012. Nurture trumps nature in a longitudinal survey of salivary bacterial communities in twins from early adolescence to early adulthood. Genome Res 22:2146–2152. doi:10.1101/gr.140608.112. 23064750 PMC3483544

[61] Brennan AT, Bonawitz R, Gill CJ, Thea DM, Kleinman M, Useem J, Garrison L, Ceccarelli R, Udokwu C, Long L, Fox MP. 2016. A meta-analysis assessing all-cause mortality in HIV-exposed uninfected compared with HIV-unexposed uninfected infants and children. AIDS 30:2351–2360. doi:10.1097/QAD.0000000000001211. 27456985

[62] Dwyer-Lindgren L, Cork MA, Sligar A, Steuben KM, Wilson KF, Provost NR, Mayala BK, VanderHeide JD, Collison ML, Hall JB, Biehl MH, Carter A, Frank T, Douwes-Schultz D, Burstein R, Casey DC, Deshpande A, Earl L, El Bcheraoui C, Farag TH, Henry NJ, Kinyoki D, Marczak LB, Nixon MR, Osgood-Zimmerman A, Pigott D, Reiner RC, Ross JM, Schaeffer LE, Smith DL, Davis Weaver N, Wiens KE, Eaton JW, Justman JE, Opio A, Sartorius B, Tanser F, Wabiri N, Piot P, Murray CJL, Hay SI. 2019. Mapping HIV prevalence in sub-Saharan Africa between 2000 and 2017. Nature 570:189–193. doi:10.1038/s41586-019-1200-9. 31092927 PMC6601349

[63] Takahashi N, Nyvad B. 2011. The role of bacteria in the caries process: ecological perspectives. J Dent Res 90:294–303. doi:10.1177/0022034510379602. 20924061

[64] Martin M. 2011. Cutadapt removes adapter sequences from high-throughput sequencing reads. EMBnet J 17:10–12. doi:10.14806/ej.17.1.200.

[65] Callahan BJ, McMurdie PJ, Rosen MJ, Han AW, Johnson AJA, Holmes SP. 2016. DADA2: high-resolution sample inference from Illumina amplicon data. Nat Methods 13:581–583. doi:10.1038/nmeth.3869. 27214047 PMC4927377

[66] Wood DE, Lu J, Langmead B. 2019. Improved metagenomic analysis with Kraken 2. Genome Biol 20:257. doi:10.1186/s13059-019-1891-0. 31779668 PMC6883579

[67] R Core Team. 2017. R: a language and environment for statistical computing. R Foundation for Statistical Computing, Vienna, Austria.

[68] Silverman JD, Washburne AD, Mukherjee S, David LA. 2017. A phylogenetic transform enhances analysis of compositional microbiota data. Elife 6:e21887. doi:10.7554/eLife.21887. 28198697 PMC5328592

[69] McMurdie PJ, Holmes S. 2013. phyloseq: an R package for reproducible interactive analysis and graphics of microbiome census data. PLoS One 8:e61217. doi:10.1371/journal.pone.0061217. 23630581 PMC3632530

[70] Oksanen J, Blanchet FG, Friendly M, Kiindt R, Legendre P, McGlinn D, Minchin PR, O’Hara RB, Simpson GL, Solymos P, Stevens MHH, Szoecs E, Wagner H. 2019. vegan: Community Ecology package (R package version 2.5–6).

[71] Liaw A, Wiener M. 2002. Classification and regression by randomForest. R News 2:18–22. https://CRAN.R-project.org/doc/Rnews/.

[72] Murphy MA, Evans JS, Storfer A. 2010. Quantifying Bufo boreas connectivity in Yellowstone National Park with landscape genetics. Ecology 91:252–261. doi:10.1890/08-0879.1. 20380214

[73] Washburne AD, Silverman JD, Leff JW, Bennett DJ, Darcy JL, Mukherjee S, Fierer N, David LA. 2017. Phylogenetic factorization of compositional data yields lineage-level associations in microbiome datasets. PeerJ 5:e2969. doi:10.7717/peerj.2969. 28289558 PMC5345826

[74] Love MI, Huber W, Anders S. 2014. Moderated estimation of fold change and dispersion for RNA-seq data with DESeq2. Genome Biol 15:550. doi:10.1186/s13059-014-0550-8. 25516281 PMC4302049

