# Supplementary material for: Impact of HIV on the Oral Microbiome of Children Living in Sub-Saharan Africa, Determined by Using an rpoC Gene Fragment Metataxonomic Approach
Source: Microbiol Spectr. 2023 Jul 10;11(4):e00871-23. doi: 10.1128/spectrum.00871-23 (PMC10434123; doi:10.1128/spectrum.00871-23)
Supplement: Supplemental file 1 — Fig. S1 to S9. Download spectrum.00871-23-s0001.docx, DOCX file, 1.2 MB [file spectrum.00871-23-s0001.docx]

The impact of HIV on the oral microbiome of children living in Sub-Saharan Africa using a *rpo*C gene-fragment metataxonomic approach

Allison E. Mann, Lauren M. O'Connell, Esosa Osagie, Paul Akhigbe, Ozoemene Obuekwe, Augustine Omoigberale, Colton Kelly, the DOMHaIN Study Team, Modupe O. Coker, and Vincent P. Richards

**Supplementary Figures:**

**Figure S1**: Distribution of samples collected for this study by tooth health category and HIV status.


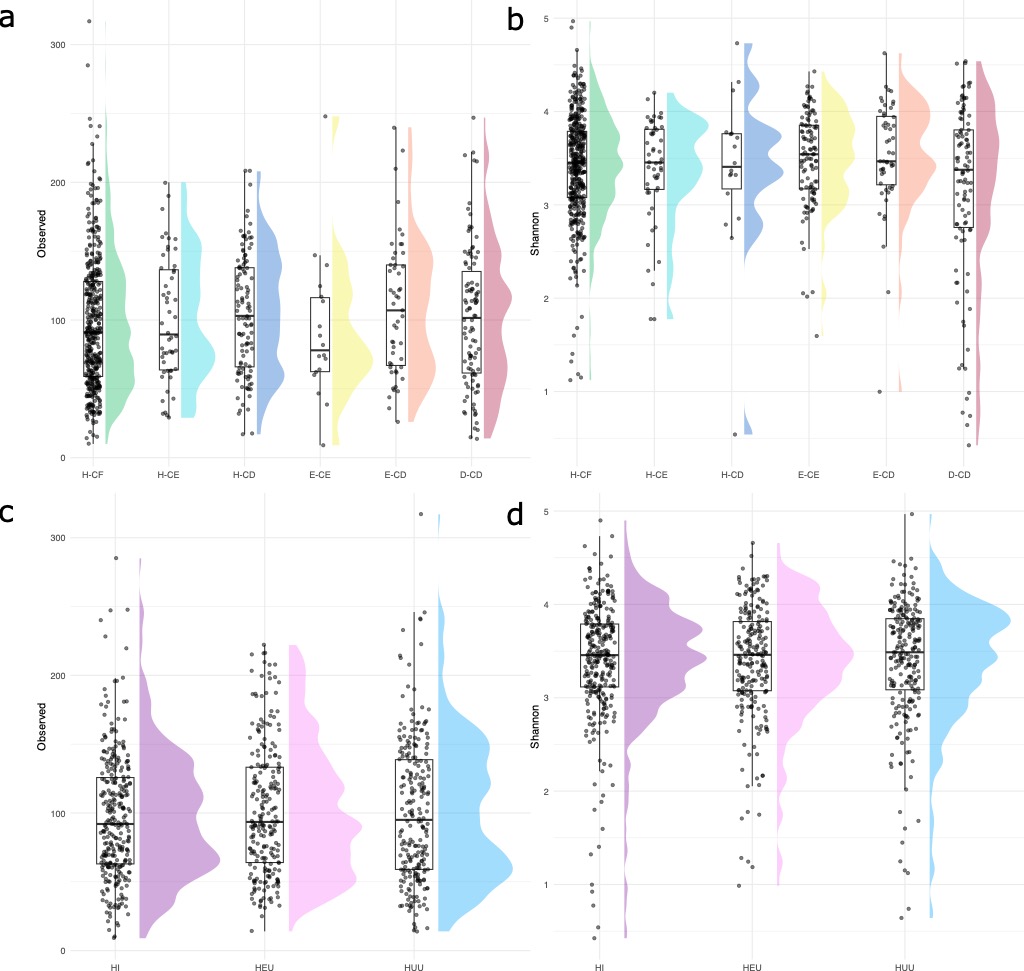


**Figure S2**: Alpha diversity (Observed ASVs and Shannon diversity) by tooth health and HIV status category. (a) Observed ASV count by tooth health category. (b) Shannon diversity by tooth health category. (c) Observed ASV count by HIV status group. (d) Shannon diversity by HIV status group. Alpha diversity was performed on rarefied data (subsampled to minimum read count across all samples).

**Figure S3**: PCoA plots of weighted UniFrac diversity across all samples colored by HIV status or tooth health. Centroids for each group visualized as larger points. Corresponding R^2^ and p values from PERMANOVA analysis using adonis2.


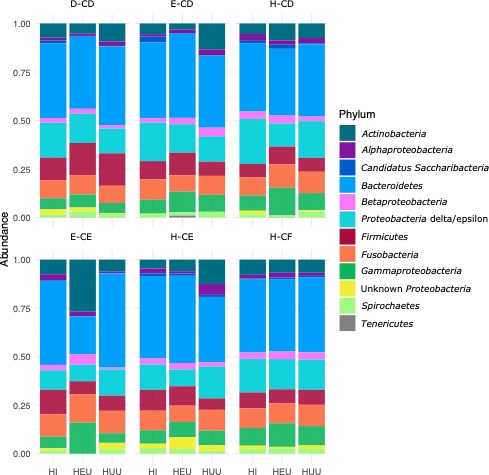


**Figure S4**: Phylum-level stacked taxonomic bar charts of different tooth health statuses split by HIV group. Each colored section of bar represents the relative abundance of a particular high-level taxonomic group averaged across all members of a HIV status group.

**Figure S5:** Frequency of different tooth health groups among age groups and alpha diversity metrics across age groups in years (approximated to the nearest year).

**Figure S6**: Relative abundance of five *Streptococcus mutans* ASVs across tooth health and HIV status. While *S. mutans* is prevalent in D-CD samples irrespective of HIV status they are not the dominant taxa in PE or PF samples. Error bars represent the standard deviation from the mean relative abundance across samples.

**Figure S7**: *Streptococcus mutans* ASVs are differentially abundant across tooth health groups. Relative abundance (log scaled) of ASVs colored by tooth health category. Stars indicate significance level of comparison; *: p <= 0.05, **: p <= 0.01, ***: p <= 0.001, ****: p <= 0.0001. Bars with dotted lines are not significant after FDR correction.

**Figure S8**: Mock community results. Fold change bar plots of the six representative oral taxa in our mock community samples using both (a) merged paired end reads and (b) forward reads only. Red dotted line indicates expected proportion across species. Error bars represent standard deviation across three replicate sequencing attempts. Raw sequencing reads were prepared in an identical manner to true samples before generating plots. Proportions lower than expected for *Streptococcus mitis* and *Schaalia odontolytica* but consistent across all species in each sequencing attempt.

**Figure S9**: Rarefaction curves generated from quality filtered data grouped by tooth health and colored by HIV status.
